# Validation of robotic resection without preoperative diagnosis for peripheral clinical T1N0 lung cancer

**DOI:** 10.1016/j.xjon.2026.101959

**Published:** 2026-07-09

**Authors:** Dao M. Nguyen, Nestor Villamiziar, Luis Miguel Cotamo, Blake Smith, Daniel Gross, Diego Avella

**Affiliations:** Division of Cardiothoracic Surgery, Thoracic Surgery Section, The DeWitt Daughtry Department of Surgery, University of Miami, Miami, Fla

**Keywords:** robotic thoracoscopy, preoperative biopsy, intraoperative biopsy, peripheral T1 lung tumor

## Abstract

**Objective:**

To evaluate safety, accuracy, and cost-efficiency of robotic thoracoscopic diagnostic wedge and immediate anatomic resection without preoperative diagnosis of peripheral nodules suspicious of cT1N0 lung cancers.

**Methods:**

We queried our robotic thoracic surgery institutional database (January 1, 2017, to December 31, 2025) to identify patients with cT1N0 tumors amenable to intraoperative wedge for cancer diagnosis and immediate curative-intent anatomic resection. Data included demographics, preoperative biopsy, operative details, postoperative outcomes, Mayo Clinic cancer score, and estimated costs associated with cancer diagnosis and resection.

**Results:**

In total, 786 patients were included in this study; 585 patients (including 65 having nondiagnostic preoperative biopsy) underwent diagnostic wedge resection: 100 (17.1%) benign and 485 (82.9%) malignant diagnosis. A total of 467 had anatomic resections (1-stage diagnosis and therapy, group 1); 266 patients had preoperative biopsy, with definite cancer diagnosis in 201 (75.8%) and 197 had anatomic resection (2-stage diagnosis then therapy, group 2), whereas 65 (24.4%) nondiagnostic biopsies required subsequent diagnostic resection, and 118 patients had wedge resection only (group 3). Three groups had similar preoperative characteristics. Larger tumors, more lobectomy, and greater incidence of nodal upstaging and costs were observed in group 2. Discordance between intraoperative and postoperative pathology was noted in 11 (2.3%) of 485 patients, with 9 having a history of carcinoma. No complications were reported in 88.6%, 83.7%, and 94.1% of patients in groups 1, 2, and 3, respectively.

**Conclusions:**

1-stage robotic resection is a safe, accurate, and cost-efficient strategy in this patient population. Preoperative biopsy is useful in patients with a previous history of carcinoma to minimize diagnostic discordance associated with under/over-treatments.

**Figure:**
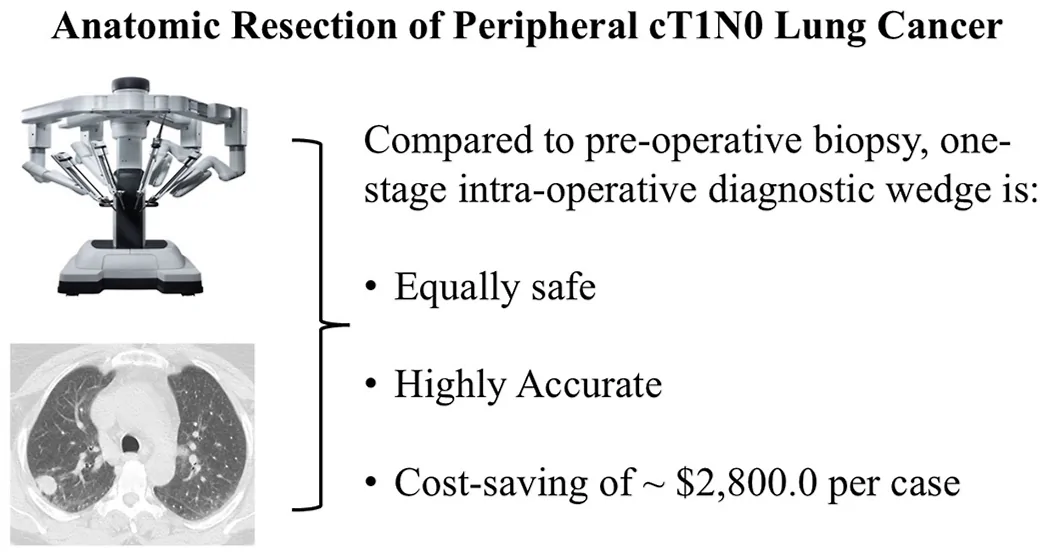
Validation of robotic thoracoscopic diagnostic wedge resection for peripheral cT1N0 tumor.

**Central Message**

Robotic thoracoscopic intraoperative diagnostic wedge and immediate anatomic resection without preoperative biopsy for peripheral clinical T1N0 lung cancer is safe, accurate, and cost-efficient.

**Perspective**

The 1-stage approach for the surgical management of peripheral cT1N0 tumors is highly accurate for cancer diagnosis with a very small risk of diagnostic discordance requiring reoperation. Bypassing preoperative biopsy would eliminate risks of procedure-associated complications, reduce patient discomfort, increase cost savings to the health system, and potentially decrease delay to curative surgery.

The treatment of choice for clinical T1N0 (cT1N0) non–small cell lung cancer (NSCLC) is anatomic resection with adequate intrathoracic lymph node staging, preferably by minimally invasive thoracoscopy surgery, without mandatory preoperative hilar/mediastinal lymph node sampling and imaging of the brain.^1^ Practice guidelines indicate either cancer diagnosis by transbronchial biopsy (TBBx) or transthoracic biopsy (TTBx) before anatomic resection (2-stage approach) or intraoperative cancer diagnosis and immediate anatomic resection of highly suspicious peripheral cT1N0 for which a minimally invasive thoracoscopy surgery wedge resection is feasible (1-stage approach).^1^ Furthermore, the 1-stage approach is indicated when preoperative tissue sampling is either nondiagnostic or not feasible because of the tumor’s location or patient’s refusal. Intraoperative benign diagnosis (false-positive preoperative presumed lung cancer diagnosis), well-described in the literature for this patient population,^2^, ^3^, ^4^ needs to be anticipated and minimized. Preresection cancer diagnosis simplifies treatment planning, patient counseling, and efficient use of operating room resources. Lung biopsy by TBB or TTBx has the risk of equivocal or false-negative results, more by TBBx without navigation,^5^ and complications, particularly with TTBx.^6^ Moreover, the cost of preoperative tissue sampling and the potential delay of surgical therapy for lung cancer cannot be overstated.

As consultants, thoracic surgeons frequently evaluate patients with solitary solid or part-solid peripheral lung nodules either already diagnosed as NSCLC or suspicious enough to warrant a surgical evaluation for definitive therapy. In the absence of clinical indications for preresection biopsy of the index lung tumors such as a recent history of cancer or hilar/mediastinal adenopathy by positron emission tomography (PET)/computed tomography (CT) or CT scan imaging studies, the practice of routinely obtaining a preoperative cancer diagnosis or upfront 1-stage operative intervention is institution-dependent. For more than 14 years, our group has adopted a more liberal approach of offering 1-stage robotic thoracoscopy diagnostic wedge resection whenever feasible for this patient population and lobectomy or segmentectomy for appropriately selected patients on the basis of tumor size, location, and characteristics, as recently validated by phase 3 clinical trials.^7^^,^^8^ The clinical and pathologic outcomes as well as financial impact of such approach have not been defined.

This retrospective single-institution study aims to evaluate the validity of the robotic thoracoscopy 1-stage approach for cancer-suspicious peripheral cT1N0 lung nodules. The primary objectives are to define the safety and accuracy of intraoperative diagnostic wedge resection, especially the incidence of benign diagnosis (false positive) and discordance of intraoperative versus final pathologic diagnosis. The secondary objective is to estimate the costs of lung cancer diagnosis and resection by either approach.

## Methods

### Patient Population

Data for this retrospective study were extracted from our prospectively maintained thoracic surgery database, with waiver of patient consent requirement by the institutional review board (number 20180827 last reviewed and approved December 20, 2024). All adult patients undergoing robotic pulmonary resection between January 1, 2017, and December 31, 2025, by 3 American Board of Thoracic Surgery–certified noncardiac thoracic surgeons were reviewed. The following patients were excluded: those with intended therapeutic wedge resection, known benign diagnosis, induction therapy, completion anatomic resection, hilar/mediastinal lymphadenopathy by imaging requiring preoperative biopsy, multiple lung tumors, tumors >3.0 cm (greatest linear dimension of entire tumor including ground-glass opacity component by CT scan), and tumors located away from outer third of visceral pleura not feasible for intraoperative diagnostic wedge resection. The following patients were included and formed the basis of this study: those with solitary T1 (≤3.0 cm) clinically suspicious for being lung cancer located within the outer third of lung and deemed feasible for intraoperative diagnostic wedge resection as independently judged by 2 surgeons (D.M.N. and N.V.), with or without preoperative biopsy of tumor by either TTBx or TBBx and anatomic lung resection (segmentectomy, lobectomy, pneumonectomy). Patients who initially were scheduled to have intraoperative diagnostic wedge but instead underwent diagnostic and therapeutic segmentectomy or lobectomy, either because wedge would distort hilar anatomy precluding safe anatomic resection or thick/fibrotic lung parenchyma not yielding to diagnostic wedge, were included. Group 1 consisted of robotic anatomic resections for intraoperative lung cancer diagnosis, including those with nondiagnostic preoperative tumor biopsy. Group 2 included anatomic resections with a preoperative diagnosis of lung cancer. Planned anatomic resection was aborted for benign diagnosis or secondary lung cancer for which wedge resection deemed therapeutic or unexpected finding of intrapleural metastasis (M1a) as a contraindication to anatomic resection. Patients undergoing diagnostic wedge resection only were included in group 3. A composite indication for appropriate upfront resection of this group was created: growing on serial CT and/or maximum standardized uptake value [SUVmax] greater than background and negative preoperative biopsy and location not amenable to preoperative TBBx or TTBx (Appendix E1). The indication for preoperative biopsy of every patient was reviewed and deemed indicated if results would change therapy, especially in the presence of a past or current diagnosis of carcinoma, at the patient's request, or inaccessibility by intraoperative wedge resection. The study was conducted and reported in concordance with the STrengthening the Reporting of OBservational studies in Epidemiology guidelines.^9^

### Data Source and Attributes

The insitutional thoracic surgery database prospectively collects from the hospital electronic medical records (EPIC) detailed clinical parameters, including but not limiting to patient demographics, types of preoperative tumor biopsy, radiographic details (tumor size, tumor types [solid, part-solid], PET scan fludeoxyglucose-18 [FDG] SUVmax), previous interval history of cancers (sarcomas, carcinomas), operative details, pathologic diagnoses, tumor-node-metastasis staging for primary lung cancer (eighth edition), number of lymph node harvested and nodal stations explored, 30-day postoperative complications (Clavien-Dindo classification), length of hospital stay, daily pain scores recorded by nursing staff as previously described,^10^ and in-hospital and postoperative analgesics dispensed (expressed as oral morphine milligram equivalent). Fillings of opioid prescriptions within 30 days of discharge were derived from the Florida's Prescription Drug Monitoring Program database.^10^

### Cost Analysis

All robotic anatomic resections received similar preoperative, intraoperative, and postoperative ancillary cares/services. We estimated the cost in achieving cancer diagnosis and resection in each group: direct costs associated with robotic stapler use, preoperative biopsies of all patients, and indirect costs of operating room and postoperative hospital stay. Details of direct/indirect costs and methods of cost analysis are outlined in the Appendix E1.

### Statistical Analysis

Data are summarized using descriptive statistics: frequencies and percentages and medians and interquartile ranges for nonparametric variables. Statistical analyses were performed using the Fisher exact test for categorical variables and the Mann-Whitney *U* test for continuous variables. Statistical analysis was performed using GraphPad Prism (version 10.4.2).

## Results

A total of 786 patients fulfilled the inclusion criteria for study. Figure 1 summarizes the workflow of these patients: 585 patients (74.4%) underwent robotic thoracoscopy for tissue diagnosis (65 had nondiagnostic preoperative biopsy and 520 never had biopsy) of which 100 (17.1%) were benign and 485 (82.9%) had cancer diagnosis but only 467 proceeded with planned anatomic resection (group 1), as 18 did not proceed as the result of contraindications. In contrast, 266 patients (25.6%) had preoperative tumor sampling, of which 201 (75.6%) were diagnostic of lung cancer and subsequently underwent intended anatomic resection with 5 (2.5%) aborted because of contraindications with 196 constituting the 2 stage group (group 2).

**Figure 1:**
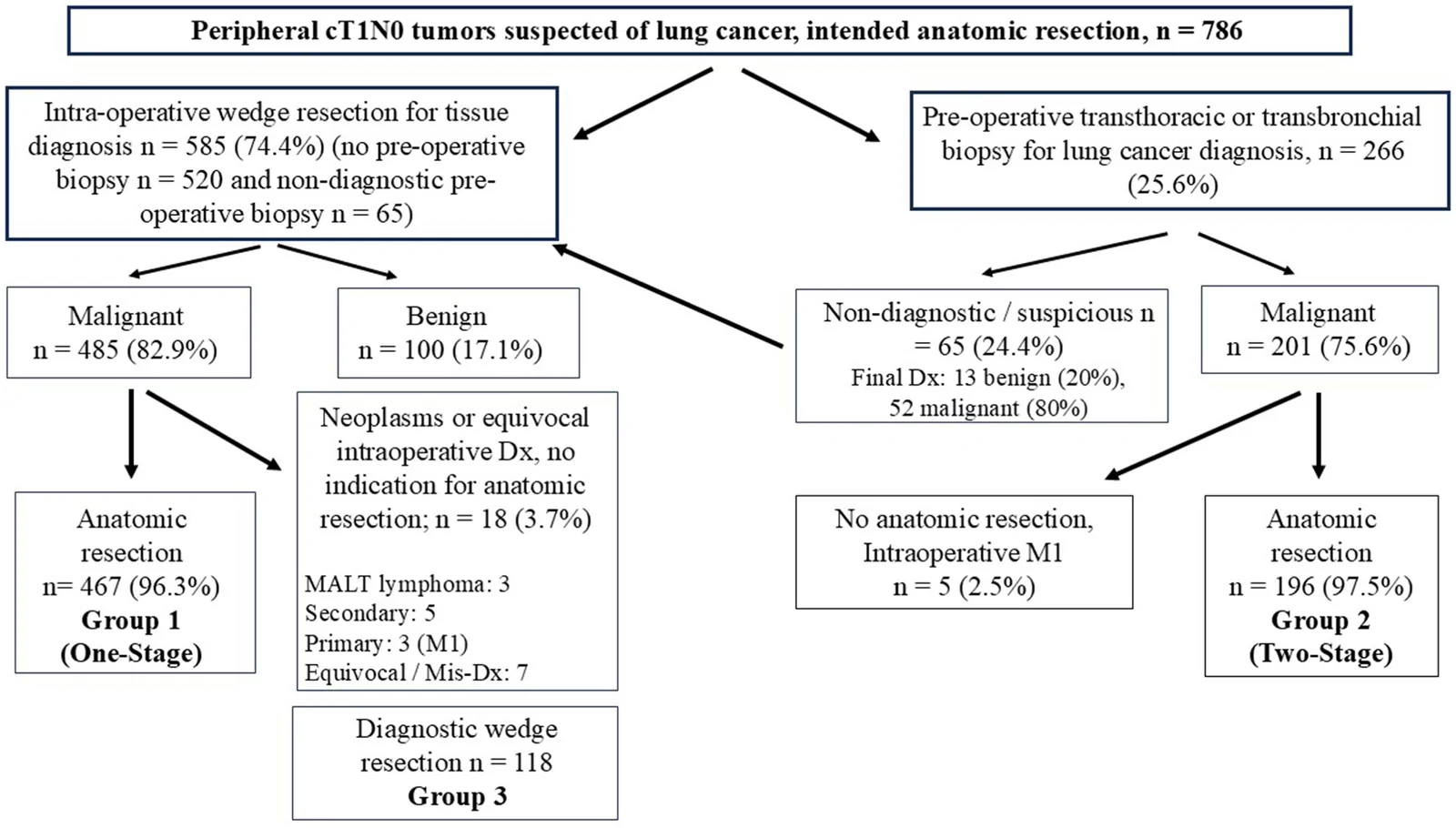
Consolidated Standards of Reporting Trials diagram of the surgical management of peripheral cT1N0 tumors. *Dx*, Diagnosis; *MALT*, mucosa-associated lymphoid tissue; *mis-Dx*, misdiagnosis.

Table 1 summarizes the preoperative characteristics of the 3 cohorts. Overall, the 3 groups were very similar in demographics and clinical characteristics. Tumors of group 2 were slightly larger than those of groups 1 and 3 (median 2.0 cm vs 1.8 cm and 1.6 cm, *P* < .0001) with maximal linear dimension of ≥2.0 cm noted in 54,1% of tumors of group 2 versus 40.7% and 15.2% tumors of group 1 and 3 (*P* = .002 vs group 1 and <.0001 vs group 3). There was no difference in FDG SUVmax between groups. Nondiagnostic preoperative biopsy was observed in 65 (24.4%) of the 266 patients by either TBBX or TTBX. Subsequent intraoperative wedge resection showed a cancer diagnosis in 52 (80.0%) and a benign diagnosis in 13 (20.0%). The Mayo Clinic scores were 78.2% [23.0-91.0] and 79.0% [53.0-92.0] in groups 3 and 1, respectively (*P* = .11).

**Table 1:** Preoperative characteristics of the 3 groups

Preoperative characteristics	One-stage approach (group 1), n = 467	*P*, group 1 vs group 2	Two-stage approach (group 2), n = 196	Intraoperative wedge (group 3), n = 118	*P*, group 1 vs group 3
Age, y, median [IQR]	70.0 [63.0-75.0]	.7633	70.0 [62.0-74.9]	67.5 [59.0-73.5]	.0106
Gender, F/M, (n,%)	267 (57.2%)/200 (42.8%)	.0565	108 (65.3%)/68 (34.7%)	78 (66.1%)/40 (33.9%)	.0935
Race (White/Black + others)	433 (92.7%)/34 (7.3%)	.2156	176 (89.9%)/20 (10.1%)	111 (94.1%)/7 (5.9%)	.6914
Ethnicity (non-Hispanic/Hispanic)	251 (53.7%)/216 (46.3%)	.0865	120 (61.2%)/76 (38.8%)	65 (55.1%)/53 (44.9%)	.8356
Comorbidity (hypertension, diabetes, cardiac, renal disorders)					
No	204 (43.7%)	.2129	100 (51.0%)	59 (50.0%)	.1611
At least 1	263 (56.3%)		96 (49.0%)	59 (50.0%)	
Smoking history (yes)	310 (66.4%)	.2129	120 (61.2%)	70 (59.3%)	.1611
FEV1 (% predicted normal)	89.0 [78.0-101.0]	.5819	91.0 [78.0-100.0]	87.0 [73.5-98.0]	.2237
DLCO (% predicted normal)	80.0 [68.0-93.0]	.0474	76.0 [65.8-91.8]	80.0 [66.0-93.0]	.9800
BMI, kg/m^2^	26.9 [24.0-30.50]	.6874	26.9 [23.8-30.9]	26.2 [21.7-30.5]	.3354
Preoperative biopsy	51		196	14	
Clinically indicated	15 (29.4%)	.7375	64 (32.6%)	7 (50%)	
Not clinically indicated	36 (70.6%)		132 (67.3%)	7 (50%)	
Size, cm	1.8 [1.3-2.3]	**<.0001**	2.0 [1.5-2.6]	1.6 [1.2-2.4]	.1230
≥2.0 cm	190 (40.7%)	**.002**	106 (54.1%)	18 (15.2%)	**<.0001**
PET/CT SUVmax	3.0 [1.8-5.2]	.0758	3.0 [2.1-6.4]	3.2 [1.8-5.1]	.5108
History of previous carcinoma	102 (21.8%)	**.0107**	62 (31.6%)	36 (30.5%)	**.0526**
Mayo Clinic score	79.0 [53.0-92.0]			78.2 [23.0-91.0]	.1138

Bold values indicate statistical significance.

*IQR*, Interquartile range; *F/M*, female/male; *FEV1*, forced expiratory volume in 1 second; *DLCO*, carbon monoxide diffusion capacity; *BMI*, body mass index; *PET/CT*, position emission tomography/computed tomography; *SUVmax*, maximum standardized uptake value.

Table 2 provides comparative analysis of operative and postoperative outcomes in groups 1 and 2. The 2 groups were very comparable, particularly length of stay, complications, pain levels, and opioid consumption. Operating time was slightly shorter in group 2 and a greater proportion lobectomy and significantly greater incidence pathologic stage 2 or 3 due to mainly lymph node upstaging.

**Table 2:** Postoperative outcomes of the 2 pulmonary anatomic resection groups

Postoperative outcomes	One-stage approach (group 1), n = 467	Two-stage approach (group 2), n = 196	*P* value
Operating time, median [IQR]/mean, min	186.0 [159.0-220.7]/196.3	173.0 [143.3-218.5]/184.0	**.0025**
Estimated blood loss, mL	50.0 [20.0-100.0]	50.0 [20.0-80.0]	.4884
Lobectomy/segmentectomy	244 (52.2%)/223 (47.8%)	127 (64.8%)/68 (34.7%)	**.0035**
Number of nodes	13.0 [10.0-17.0]	13.0 [10-16]	.1917
Number of node stations	5.0 [5.0-6.0]	6.0 [5.0-6.0]	.2109
Final pathologic diagnosis, n (%)			
Benign/neoplasm NOS	6 (1.3%)	0	
Malignant	461 (98.7%)	196 (100%)	
Secondary, n (%)	24 (5.2%)	4 (2.0%)	.0893
Primary, n (%)	437 (94.8%)	192 (98.0%)	
Stage 1, n (%)	395 (90.4%)	157 (81.8%)	**.0035**
Stage 2-3, n (%)	42 (9.6%)	35 (18.2%)	
T upstaging	12 (2.7%)	8 (4.2%)	.3109
Nodal upstaging	30 (6.9%) (19 N1; 11 N2)	27 (14.0%) (15 N1; 12 N2)	**.0061**
30-d complication, n (%)			
0	414 (88.6%)	164 (83.7%)	.0974
1-2	44 (9.4%)	25 (12.8%)	
3-4	9 (1.9%)	6 (3.0%)	
5	0	1 (0.5%)	
Length of hospital stay, d	2.0 [1.0-3.0]	2.0 [1.0-3.0]	.3174
Pain			
POD0	4.6 [3.3-5.7]	5.0 [4.0-6.5]	**.0034**
POD1	2.5 [1.2-4.1]	2.5 [1.4-4,5]	.6943
On day of discharge	1.6 [0.0-3.0]	1.6 [0.0-3.4]	.9700
MME (in-hospital + 30-d post D/C)	70.5 [15.1-180.0]	64.0 [13.7-225.7]	.6700

Bold values indicate statistical significance.

*IQR*, Interquartile range; *NOS*, not otherwise specified; *POD*, postoperative day; *D/C*, day of discharge; *MME*, morphine milligram equivalent.

Table 3 summarizes the outcomes of group 3. Of 118 patients having had diagnostic wedge resection but not proceeding to anatomic resection as initially planned, 100 were attributable to a benign diagnosis. Overall, 68% of granulomas (59/86) were submitted for culture and 17 (28.8%) were positive for a pathogen (6 fungus and 9 atypical mycobacterium species). On the basis of the retrospective composite clinical/radiographic indications for upfront resection in group 3, 22 (18.6%) resections were potentially avoidable (Table 3 and Appendix E1).

**Table 3:** Postoperative outcomes of wedge resections only

	Intraoperative wedge only (group 3) n = 118
Postoperative outcomes	
Final pathologic diagnosis, n (%)	
Benign/neoplasm NOS	100 (84.7%): 9 hamartoma/86 granuloma/5 fibrosis
Malignant: secondary/primary/MALT lymphoma	18 (15.2%): 5/3 (T1NxM1)/7
Discordant intraoperative/postoperative path Dx	7
30-d complications (Clavien-Dindo), n (%)	
0	111 (94.1%)
1-2	4 (3.4%)
3-4	3 (2.5%)
LOS, d, median [IQR]	1. 0 [1.0-2.0]
Pain	
POD0/POD1/on day of discharge	4.2 [3.0-5.5]/2.0 [0.8-3.6]/1.8 [0.4-2.9]
MME (in-hospital + 30-d postdischarge)	29.7 [5.3-197.3]
Culture	59 of 86 granulomas (68.6%)
No growth/positive for pathogens	42 (71.2%)/17 (28.8%): 6 fungus, 9 atypical TB
Factors affecting decision to offer surgical biopsy	
1. Serial CT scans/with evidence of growth	82/34
2. FDG-SUVmax > background blood pool	82
3. Locations not amenable to preoperative TBBx or TTBx	40
4. Nondiagnostic preoperative TBBX or TTBx	14
Composite indications for resections	
1 and/or 2 and 3 and 4	96 (81.3%)
None: unnecessary resection	22 (18.6%)

*NOS*, Not otherwise specified; *MALT*, mucosa-associated lymphoid tissue; *Dx*, diagnosis; *LOS*, length of stay; *IQR*, interquartile range; *POD*, postoperative day; *MME*, morphine milligram equivalent; *CT*, computed tomography; *FDG*, fludeoxyglucose-18; *SUVmax*, maximum standardized uptake value; *TBBx*, transbronchial biopsy; *TTBx*, transthoracic biopsy.

Overall, of 585 patients receiving a 1-stage approach, 100 had a benign diagnosis, with 100% concordance with final pathologic report, for a false-positive incidence of 17.1%. Of 485 cancer diagnoses, there were 11 cases (11/485; 2.3%) of discordant intraoperative frozen-section and final postoperative pathologic report (Table E1). Five of these patients did not need re-resection because of adequate margins for small typical neuroendocrine neoplasms or multifocal adenocarcinomas whereas 2 (2/485, 0.4%) were undertreated and required re-resection as completion lobectomy and 4 were overtreated, as these secondary cancers could have been adequately managed with therapeutic wedge resection only. Intraoperative wedge had a diagnosis rate for neoplasms of 97.7% (474/485). This compares favorably with only 75.6% (201/266) cancer diagnosis of preoperative biopsy due to 24.4% (65/266) nondiagnostic/equivocal results.

**Table E1:** Summary of 11 cases of pathologic discordance observed in group 1

Patient	Previous history of carcinoma	Intraoperative diagnosis	Surgical procedure	Final pathology diagnosis	Follow-up surgical procedure	Comment on first operation
1	Endometrium, > 20 y; Lung, 5 y	Lung adenocarcinoma	Lingular segmentectomy	Endometrial adenocarcinoma	None	Potentially overtreated
2	Uterine cervix, 5 y	Lung adenocarcinoma	Right upper lobectomy	Uterine cervix adenocarcinoma	None	Potentially overtreated
3	Parotid gland, 5 y	Lung adenocarcinoma	Right upper lobectomy	Parotid adenocarcinoma	None	Potentially overtreated
4	No	Neuroendocrine tumor	Lower lobe superior segmentectomy	MALT lymphoma	None	Potentially overtreated
5	Breast, 6 y	Breast cancer metastasis	Wedge	Lung adenocarcinoma	Completion lobectomy	Undertreated
6	Breast, 3 y	Equivocal adenocarcinoma	Wedge	Invasive lung adenocarcinoma	Completion lobectomy	Undertreated
7	No	Equivocal tissue diagnosis	Wedge	Typical neuroendocrine tumor	none (tumor = 1.1 cm)	Acceptable, margin >1.0 cm
8	Breast, 5 y	Breast cancer metastasis	Wedge	Typical neuroendocrine tumor	None (tumor = 1.2 cm)	Acceptable, margin >1.0 cm
9	Kidney chromophobe, 5 y	Renal cell cancer	Wedge	Typical neuroendocrine tumor	None (tumor = 1.5 cm)	Acceptable, margin >1.0 cm
10	Stage 4 renal cell cancer ongoing therapy, 1 y	Renal cell cancer	Wedge	Adenocarcinoma ×2 (T3N0)	None, adjuvant systemic therapy	Acceptable as borderline PFT for anatomic resection
11	Melanoma, 3 y	Melanoma metastasis	Wedge	Typical neuroendocrine tumor	None (tumor = 0.7 cm)	Acceptable, margin >2.0 cm

*MALT*, Mucosa-associated lymphoid tissue; *PFT*, pulmonary function test.

Differential costs of cancer diagnosis and resection are shown in Figure 2. Total tangible costs of group 2 were greater (median [interquartile range]: $13,700.5 [12,102.0-15,624.5]/mean: $14,884.3) than those of group 1 ($11,106.0 [8899.0-13,433.0]/mean: $11,845.9, *P* < .0001) for a mean difference of $3038.4/case. Further analysis of cost differentials of groups 1 and 2 with factoring in the financial burden of 22 avoidable operations in group 3 is detailed in the Appendix E1, and this reduced the cost difference from $3038.4 to $2787.0/case.

**Figure 2:**
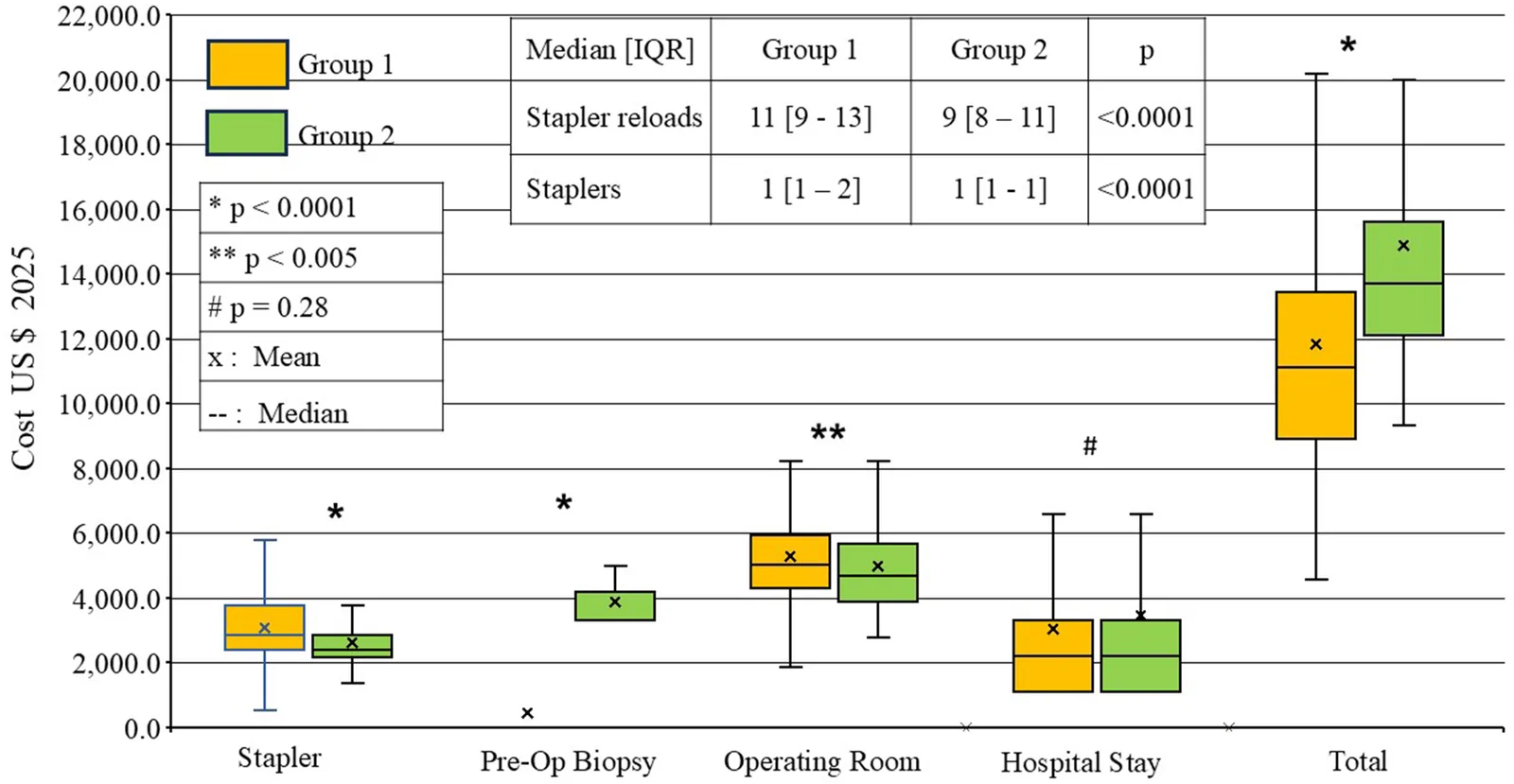
Comparative analysis of cost associated with cancer diagnosis and resection. *IQR*, Interquartile range.

## Discussion

This single-institution retrospective analysis demonstrates that intraoperative diagnostic resection for suspicious peripheral cT1N0 lung cancer is highly accurate, with diagnostic yield of 98.1% (concordant intraoperative and postoperative diagnosis of 100% for benign and of 97.7% for neoplasms), safe with 88.6% to 94.1% no 30-day complications without operative mortality and cost-efficient with an average reduction of approximately US $2800.0 per case of costs associated with cancer diagnosis and definitive therapy with anatomic resection.

The use of imaging of the chest for ever-expanding clinical indications is associated with increasing detection of incidental T1 nodules. The initial management of these nodules by pulmonary physicians, medical oncologists, or thoracic surgeons is frequently guided by management algorithms set forth by the Fleischner Society^11^ or the Lung-RADS classification system.^12^ For the subset of peripheral T1N0 lung tumors suspicious of malignancy (interval growth, irregular spiculated border, transformation/progression to part-solid ground-glass opacity, high PET/CT FDG-SUVmax) and amenable to diagnostic wedge resection, the decision to obtain a preoperative tissue diagnosis of cancer before thoracic surgery consultation without preoperative biopsy is provider- and institution-dependent. Surgical management of biopsy-proven cT1N0 is straightforward regarding patient counseling, surgical planning, and predictable use of operating room resources. However, not all peripheral cT1N0 tumors require a preoperative biopsy, especially if the clinical suspicion is so high that an equivocal or nondiagnostic biopsy would not negate the initial recommendation for surgical resection. We adopt a more liberal approach with 1-stage intervention for peripheral cancer-suspected cT1N0, as evidenced by 74.4% of such patients who underwent lung resection without a preoperative diagnosis. Using our own algorithm of patient stratification to preoperative biopsy or upfront surgery, 67% to 70% of preoperative biopsies were deemed “not clinically indicated.” Much cost-savings could have been achieved had these patients undergone upfront resections. We always counsel our patients about the observed 17% rate of benign diagnosis as risk-benefit discussion before surgical intervention. We have performed a prospective biannual data audit since 2020 and observed a consistent 15% to 17% incidence of benign intraoperative diagnosis. An operation that results in a benign diagnosis should not be construed as unnecessary. 1-stage diagnostic/therapeutic surgical intervention is the most reasonable option for patients with serial interval CT scans and/or tumors in location not amenable to TTBx or TBBx and high Mayo Clinic malignancy risk score. We reviewed our group 3 preoperative notes and identified clear documentation of only 14 instances (14/118 = 11.9%) of patient requesting upfront resection in the preoperative visits. Such patient input must have been very compelling to be included in the note. Because this is a surgical database, we could not ascertain the number of cT1N0 nodules that continued surveillance or quantify instances that surgical decision was postponed after multimodality discussion with our medical oncologists and/or pulmonologists. It is important to minimize postoperative complications and pain/opioid use in all patients who undergo thoracic surgery, especially group 3, as reported in this study (Table 3). Another factor one must consider is the diagnostic yield, complications, and time delay in obtaining a preoperative biopsy with intervention radiology or interventional pulmonology unless the surgeons perform transbronchial biopsy as part of their practice. Our study straddled the time when robotic navigational bronchoscopy platforms (ION [Intuitive Surgical] or Monarch [Johnson & Johnson MedTech]) was introduced to clinical practice around May 2022. With increasing diagnostic accuracy and low complication rates, navigational TBBx will replace TTBx as the procedure of choice for lung tumor biopsy.^6^ More peripheral cT1N0 would likely have navigational TBBx before definitive anatomic resection. In fact, since January 1, 2023, TBBx (two thirds of which was ION navigational bronchoscopy) accounted for 60% of preoperative biopsy versus 21% (10% ION) before that date of group 2 patients. It is noteworthy that 80% of nodules with nondiagnostic preoperative biopsies were eventually proven to be malignant, similar to a previous report.^13^ Delayed curative surgical therapy for early-stage NSCLC is associated with suboptimal overall survival.^14^ Whether 2-stage management of peripheral cT1N0 is associated with the prolongation of time between first imaging study identifying suspicious tumors and definitive therapy when compared with the 1-stage approach remains to be determined. We did see significantly greater nodal upstaging in group 2 versus group 1. It is the current focus of our ongoing research to determine the factors associated with this difference.

Although 17% benign intraoperative diagnosis is similar to previous reports,^2^, ^3^, ^4^ it is still high. The development of a better algorithm to minimize the resection of benign lung pathology is clearly needed. By applying the composite surgical indications for upfront resection, as shown in Table 3, we could potentially reduce unnecessary operations by 18% or greater in our practice. The composition of benign diagnosis (granuloma, lung fibrosis, hamartoma) identified in our patient population was similar to those reported in the literature.^2^, ^3^, ^4^ The high incidence of lobectomy as well as nodal upstaging in group 2 was attributable to significantly greater number of tumors ≥2.0 cm (T1c) in this cohort, and these are all associated with increased incidence of N1/2 metastasis.^15^^,^^16^ The 14% incidence of nodal upstaging in group 2 is similar to that reported by Potter and colleagues^17^ when they queried the Society of Thoracic General Thoracic Surgery Database. It is possible that larger tumors were more likely to be referred for preoperative biopsy. Nine of 11 patients (81.8%) with discordant pathologic diagnosis had a history of carcinoma; preoperative biopsies and appropriate tumor marker analysis might have mitigated intraoperative misdiagnosis and streamlined surgical planning and the magnitude of lung parenchyma resection commensurate with cancer type, size, and location.

Our study has many limitations. There were uncontrollable biases inherent to a retrospective clinical study. This study reflects our institutional practice and is not readily generalizable. We could not provide accurate complications associated with preoperative tumor biopsy because many were performed at outside facilities. Because the study population is limited to those who underwent surgical intervention, we do not have information about the accuracy of preoperative transthoracic and transbronchial biopsies, specifically the number of patients that obtained a benign diagnosis and never required surgery. Our database does not contain patient-reported outcomes to assess the positive impact of benign diagnosis on patients such as relief of anxiety and no further need for surveillance with CT scans in addition to identification of pathogen on culture. It was not possible to perform granular analysis of true costs of global lung cancer care as many preoperative biopsies were performed elsewhere and inability to access institution finances for accurate case expenses.

## Conclusions

This single-institution retrospective analysis demonstrated that single stage intraoperative diagnosis/therapy for peripheral suspicious cT1N0 tumor, when compared with the 2-stage approach, is safe, highly accurate with an extremely low likelihood of reoperation for completion lobectomy and cost-effective. Preoperative biopsy, whenever feasible, should be performed for patients with history of carcinomas to reduce risk of intraoperative pathologic discordance and to optimize surgical planning.

### Webcast

You can watch a Webcast of this AATS meeting presentation by going to: https://www.aats.org/resources/validation-of-wedge-followed-b-12375.

**Figure:**
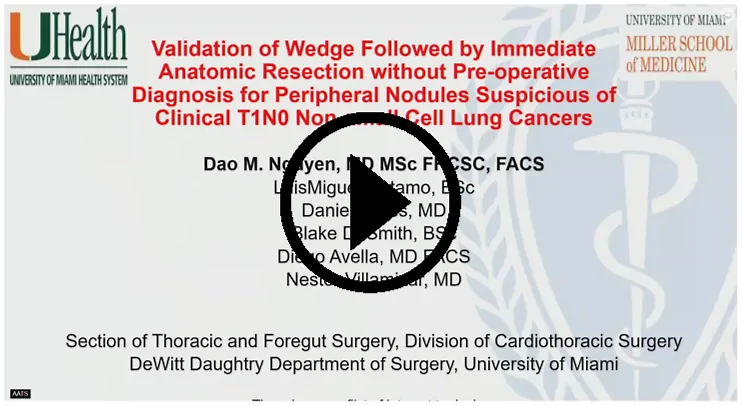

### Audio

You can listen to the discussion audio of this article by going to the supplementary material section below.

## Conflict of Interest Statement

The authors reported no conflicts of interest.

The *Journal* policy requires editors and reviewers to disclose conflicts of interest and to decline handling or reviewing manuscripts for which they may have a conflict of interest. The editors and reviewers of this article have no conflicts of interest.

## Appendix E1. Cost Analysis

The current 2025 cost of stapler (SureForm 45-mm curved tip 48045; Intuitive Inc) was US$ 520/unit and of stapler reloads (reload SureForm 45 mm 48345W/B/G/B; Intuitive Inc) was US$ 210/unit (University of Miami Health System Central Supply). The approximate reimbursements of biopsy procedures (2025 national average Medicare quotes: professional fee and facility fee; https://www.cms.gov/medicare/physician-fee-schedule/search. Accessed March 1, 2026) were used as surrogates of costs: transthoracic biopsy (TTBx) (Current Procedural Terminology [CPT] code 32408): US $3300.0; transbronchial biopsy (TBBx) with endoscopic bronchial ultrasound (CPT 31628 and 31654): US $4200.0; TBBx with endoscopic bronchial ultrasound and navigational guidance (CPT 31628, 31654 and 31627): US$ 5200.0. Indirect per diem hospital admission cost was $1100.0 and operating room cost was $1550.0/hour (University of Miami Health System Financial Department).

One of the concerns was the extra cost accrued by performing some potentially avoidable operations in group 3 in the process of making cancer diagnosis by upfront diagnostic wedge resection. In addition to the cost comparison between group 1 and group 2 as described in the Methods section, the following cost analysis was carried out to address the financial burden of performing 22 potentially unnecessary operations in group 3, as indicated in Table 3. The additional cost to achieve lung cancer diagnosis by the 1-stage approach was defined as costs of extra stapler (average 2 robotic stapler reload per case) and additional operating time of group 1 (average 13 minutes) together with the financial burden of 22 unnecessary operations (identified by the composite clinical/radiographic indications for upfront resection) of group 3 as suggested by peer-review. The total cost of 2 extra stapler uses (n = 467) was $196,140.0 (A), the cost of an extra 13 minutes of operating room time (n = 467) was $163,917.0 (B) of group 1 and total cost of 22 “avoidable/unnecessary” cases (robotic stapler cost, operating room and hospital stay) was $143,506.0 (C). The total additional cost of the 1-stage approach was (A + B + C) $503,563.0, and the average additional cost was (A + B + C/467) $1078.3. The upfront average cost of preoperative biopsy of group 2 was $3865.3 (before accounting for intraoperative and hospital stay cost). The average additional cost of group 1 to achieve intraoperative diagnosis, factoring the financial burden of unnecessary operation, was $1078.3. The cost differential to achieve lung cancer diagnosis between the 2 groups now was $2787.0 ($3865.3-$1078.3). Before factoring additional cost, the average cost differential between groups 1 and 2 was $3,038.4 (Figure 1 and Results) for a net difference of $251.4/case ($3038.4-$2787.0). Statistical analysis was not feasible secondary to the method this form of cost comparison was made. By either analysis, group 1 was still more cost-effective (net saving of $2787.0 to $3038.4 per case).

## Retrospective Analysis of Indications for Resection of Group 3

Granular review of clinical and radiographic data was performed to define indications for upfront resection of this group with benign diagnosis. The following are considered appropriate indications for surgical intervention: growth on serial computed tomography scans, fludeoxyglucose-18/maximum standardized uptake value above blood pool background activity, nondiagnostic preoperative biopsy (indicative of sufficiently suspicious of malignancy for intervention), and locations of lung nodules not amenable to neither TBBX nor TTBx. A composite indication for appropriate upfront resection of patients in group 3 was created: growing on serial computed tomography and/or fludeoxyglucose-18/maximum standardized uptake value greater than background and negative preoperative biopsy and location not amenable to preoperative TBBx or TTBx. Table 3 summarizes this analysis. Overall, 96 (81.4%) patients fulfilled these composite criteria for surgical tissue sampling, 22 (18.6%) patients did not, who were considered as having had unnecessary operations. These granular data are now included in Table 3. The median Mayo Clinic score of this subgroup was 12.0% (vs 78.2% of the entire cohort), and all lung fibrosis/hamartoma final diagnosis and some necrotizing/non-necrotizing granulomas were in this subgroup. Excluding these 22 patients from the group 1 cohort (585−22 = 563) would reduce the incidence of benign surgical biopsy (100−22 = 78) (false-positive rate) to 13.8% (78/563).

## Supplementary Data

### Audio

Audio 1 This is an audio recording of the presentation of the associated abstract and discussion. The abstract presentation ends at 7:30 and the discussion begins at 7:42.

Video 1 Video available at: https://www.jtcvs.org/article/S2666-2736(26)00382-7/fulltext.
